# Immunological larval polyphenism in the map butterfly *Araschnia levana* reveals the photoperiodic modulation of immunity

**DOI:** 10.1002/ece3.4047

**Published:** 2018-04-19

**Authors:** Arne Baudach, Kwang‐Zin Lee, Heiko Vogel, Andreas Vilcinskas

**Affiliations:** ^1^ Institute for Insect Biotechnology Justus Liebig University Giessen Germany; ^2^ Max‐Planck Institute for Chemical Ecology Jena Germany; ^3^ Department Bioresources Fraunhofer Institute for Molecular Biology and Applied Ecology Giessen Germany

**Keywords:** antimicrobial peptides, *Araschnia levana*, immunity, metamorphosis, phenotypic plasticity, polyphenism

## Abstract

The bivoltine European map butterfly (*Araschnia levana*) displays seasonal polyphenism characterized by the formation of two remarkably distinct dorsal wing phenotypes: The spring generation (*A. levana levana*) is predominantly orange with black spots and develops from diapause pupae, whereas the summer generation (*A. levana prorsa*) has black, white, and orange bands and develops from subitaneous pupae. The choice between spring or summer imagoes is regulated by the photoperiod during larval and prepupal development, but polyphenism in the larvae has not been investigated before. Recently, it has been found that the prepupae of *A. levana* display differences in immunity‐related gene expression, so we tested whether larvae destined to become spring (short‐day) or summer (long‐day) morphs also display differences in innate immunity. We measured larval survival following the injection of a bacterial entomopathogen (*Pseudomonas entomophila*), the antimicrobial activity in their hemolymph and the induced expression of selected genes encoding antimicrobial peptides (AMPs). Larvae of the short‐day generation died significantly later, exhibited higher antibacterial activity in the hemolymph, and displayed higher induced expression levels of AMPs than those of the long‐day generation. Our study expands the seasonal polyphenism of *A. levana* beyond the morphologically distinct spring and summer imagoes to include immunological larval polyphenism that reveals the photoperiodic modulation of immunity. This may reflect life‐history traits that manifest as trade‐offs between immunity and fecundity.

## 1 INTRODUCTION

Polyphenism is defined as the environmentally induced development of distinct alternative morphs encoded by the same genome (Shapiro, 1976) and plays an important role in evolutionary theory. Seasonally changing environmental parameters can in some species result in seasonally occurring phenotypes. This so‐called seasonal polyphenism is widely distributed among plants and animals (Nijhout, 2003) and has been postulated to produce phenotypes which are better adapted to a forthcoming environment, but the adaptive significance of seasonal morphs is still under debate (Simpson, Sword, & Lo, 2011). The mechanisms behind this phenomenon are poorly understood. However, its significance for sexual selection, adaptive evolution, and speciation represents as research focus in the emerging field called EcoEvoDevo (Beldade, Mateus, & Keller, 1984). Immunity represents a complex trait which has not been considered in this context. Innate immunity of insects lacks the antibodies‐based memory known from vertebrates and relies mainly on cellular mechanisms such as phagocytosis or multicellular encapsulation of invading pathogens or parasites and humoral defenses among which antimicrobial peptides play a predominant role (Mylonakis, Podsiadlowski, Muhammed, & Vilcinskas, 2016).

The European map butterfly *Araschnia levana* (Linnaeus, 1758) (Nymphalidae: Nymphalinae) is a bivoltine, diphenistic species that has been studied extensively for more than a century as an example of seasonally induced phenotypic plasticity with respect to both its proximate and ultimate causes (Fischer, 1907; Fric, Konvička, & Zrzavy, 2004; Kratochwil, 1980; Müller, 1960; Vilcinskas & Vogel, 2016). Seasonal polyphenism manifests in the formation of two remarkably distinct dorsal wing phenotypes: the spring generation (*A. levana levana*), predominantly orange with black spots, which develops from diapause pupae, and the summer generation (*A. levana prorsa*), with black, white and orange bands, which develops from subitaneous pupae. The proximate cause of this polyphenism and its physiological foundation have been thoroughly investigated. The developmental trajectory (diapause/nondiapause) depends on the photoperiod (Müller, 1955, 1956). If larvae receive less than 8 hr of daylight, they exclusively develop into diapause pupae and consequently the spring *levana* form, whereas more than 16 hr of light triggers obligate subitaneous development which, following a pupal stage lasting ~2 weeks, results in the summer *prorsa* form. The molecular switch between the two routes is hormonally controlled by the timing of ecdysteroid release, which in turn is determined by day length (Koch, 1992, 1996; Koch & Bückmann, 1987). When adult development is triggered by 20‐hydroxyecdysone 2 days after pupation, the pupa is said to be summer‐primed (derived from a long‐day larva) and the summer *prorsa* form develops, whereas if the 20‐hydroxyecdysone peak occurs 5 days after pupation, the pupa is said to be spring‐primed (derived from a short‐day larva) and the spring *levana* form develops. In addition to the obvious color polyphenism, several other more subtle characteristics differ among the two variants. The summer form has a larger wing area, more rounded wing tips and a lower wing load, but also a heavier thorax, a lower abdomen‐to‐body‐mass ratio, higher mobility, and a more open population structure (Fric & Konvička, 2000; Fric & Konvička, 2002). There are various hypotheses regarding the adaptive significance of the seasonal variations in this species, but few studies have provided experimental validation. Generally, lepidopteran wing color polyphenisms have been attributed to seasonally varying climatic conditions, such as wet or dry seasons in the tropics, or temperature shifts in temperate regions (Brakefield, 1996; Brakefield & Larsen, 1984; Watt, 1968 Windig, Brakefield, Reitsma, & Wilson, 1994). An alternative or complementary theory links predator avoidance to plastic color patterns, and the validity of this concept has been demonstrated in some species, but not in *A. levana* (Stevens, 2005). A phylogenetic analysis of polyphenism in *A. levana* largely rejected the thermoregulation hypothesis because the origin of the two phenotypes likely precedes the dispersal of the species into Palearctic regions (Fric et al., 2004). Predator avoidance is also unlikely to be the major selective pressure explaining the two wing color phenotypes because both forms were attacked at the same frequency in a field study (Ihalainen & Lindstedt, 2012). It seems likely that color polyphenism in insects reflects the interaction of multiple selection pressures given that coloration has various functions and is linked to morphology, life history, and development in *A. levana*. Interestingly, a recent transcriptomic analysis indicated that the phenotypic differences in the map butterfly correlate with strong differential gene expression in a number of different gene families (Vilcinskas & Vogel, 2016). Among these were genes related to cuticle formation (overwintering) and nutrient reservoir activity (accelerated metamorphosis) which seems intuitive, but also genes that regulate innate immunity—a finding which is more difficult to interpret. The immune system has a profound beneficial impact on the fitness of a species but constant activity causes the depletion of resources (Schwenke, Lazzaro, & Wolfner, 2016). Innate immunity is therefore a trait that is likely to be under constant selective pressure, the manifestation of which can potentially have unforeseeable pleiotropic effects. Neither the map butterfly's immune system nor its juvenile stages have yet received much attention, and there have been no studies focusing on both aspects under a realistic attack situation. In addition, it is difficult to determine the adaptive value of a trait when only studying a single life stage. Given the differences in gene expression in the prepupae as described by Vilcinskas and Vogel (2016), we therefore set out to test the novel hypothesis that transcriptomic differences in *A. levana* larvae can translate into photoperiod‐dependent differences in innate immunity when challenged by an entomopathogen. We measured the survival of long‐day and short‐day larvae when attacked by *Pseudomonas entomophila*, examined the bacterial clearance rates in the hemolymph, and surveyed the expression levels of four genes encoding antimicrobial peptides (AMPs). We selected three members of AMP families which are specific for Lepidoptera (attacin, lebocin, and gloverin) as revealed in a recent comparative analysis of insect genomes and transcriptomes (Mylonakis et al., 2016).

We report, for the first time, polyphenic innate immunity in *A. levana* larvae and also characterize the different immunity‐related phenotypes induced by two priming regimes representing summer and autumn day lengths. We discuss our findings in the context of life‐history theory and in relation to the well‐studied adult polyphenism.

## 2 MATERIALS AND METHODS

### 2.1 Rearing

Prior to rearing under controlled laboratory conditions, adult *A. levana prorsa* butterflies were collected in forests around Albach and Lich, Hesse, Germany (approximately 50°32′30.2″N 8°49′12.2″E) during the summer of 2016. The butterflies were transferred into indoor gauze cages (0.95 × 0.6 × 0.6 m) fitted with multiple potted stinging nettle plants (*Urtica dioica*) at 22°C and ~60% relative humidity. If the humidity fell below 50%, the cages were manually sprayed with water. The butterflies were given ad libitum access to 20% sugar solution provided in sponges attached to the cage walls, and the sponges were sprinkled daily with the solution to ensure a constant food supply. To induce subitaneous development in 100% of the offspring, the maximum amount of natural summer daylight (~17 hr at the summer solstice) was exceeded by imposing a 20‐h photoperiod using a Bright Sun ULTRA Desert 150 W gas‐discharge lamp (Exo Terra) complemented with a standard 40 W infrared to provide an additional mating stimulus (long‐day conditions). To induce diapause development in 100% of the offspring, an 8‐h photoperiod was imposed (short‐day conditions). After females had mated and oviposited their eggs as typical vertical strings (egg towers) on the underside of nettle leaves, the larvae were allowed to hatch on the plants before they were transferred to smaller rearing cages (0.5 × 0.5 × 0.5 m) containing multiple *U. dioica* plants. The larvae were fed ad libitum on the nettles, which were replaced when most of the leaves had been consumed. After the completion of all five larval stages and the pupal phase, the adult butterflies were transferred back to the flight cages for reproduction.

### 2.2 Treatment

On the first day after the fourth larval molt (fifth instar, L5D1), larvae from both the long‐day and short‐day groups (total sample size was *n* = 240, *n* = 30 per photoperiod regime and assay) were injected with 4 μl *P. entomophila* GFP strain (10^8^ cells/μl) kindly provided by Prof. Dr. Bruno Lemaitre (École Polytechnique Fédérale de Lausanne) (Mulet, Gomila, Lemaitre, Lalucat, & García‐Valdés, 2012) or 4 μl PBS as a control. Survival was recorded until pupation, and pupal development was tracked for 3 weeks to ensure the process was normal. All resulting adults developed according to their photoperiodic priming. We also measured bacterial clearance after administering a sublethal dose of *P. entomophila* (10^7^ cells/μl) by sampling 5 μl of hemolymph 3, 6, 9, 12, 24 and 48 hr postinjection from separate sets (*n* = 5) of larvae. Briefly, larvae were dorsolaterally punctured with a micro‐injector capillary containing 2 μl of mineral oil to prevent contact between the hemolymph and the air, which would induce the melanization reaction. The hemolymph was then withdrawn from the hemocoel and transferred into pre‐cooled Eppendorf tubes containing 35 μl PBS. The samples were stored on ice and serially diluted in PBS before pipetting onto LB agar plates (supplemented with 100 μg/ml rifampicin for selection). The first dilution field with clearly distinct and GFP‐labeled colonies was then used to count clones under a Leica MZ16 F fluorescence stereomicroscope allowing us to calculate the number of freely circulating *P. entomophila* cells (per microliter hemolymph). After the hemolymph was extracted, the larvae were flash frozen in liquid nitrogen and stored at −80°C for subsequent gene expression analysis. The following genes encoding members of the following four lepidopteran‐specific AMP families (Mylonakis et al., 2016) were assessed: lebocin, attacin, hemolin, and gloverin.

### 2.3 Gene expression analysis

The expression levels of relevant AMP genes were measured at the six sampling time points by quantitative real‐time PCR. Briefly, total RNA was isolated using the PeqLab peqGOLD MicroSpin total RNA Kit, and the quantity and purity of the samples were assessed using a NanoDrop spectrophotometer (PeqLab). First‐strand cDNA was synthesized using the First‐Strand cDNA Synthesis Kit according to the manufacturer's protocol (Thermo Fisher Scientific). Primers for real‐time PCR were designed using Primer3 software (Untergasser et al., 2012), and available primer pairs were selected based on the lowest number of potential self‐annealing structures and primer loops. Gene‐specific primers were designed based on nucleotide sequences derived from previously published RNA‐Seq data (Vilcinskas & Vogel, 2016) (Table S1). The ribosomal protein L10 gene (*RPL10*) was used for normalization and PBS controls served as calibrator. Quantitative real‐time PCR was conducted using an Applied Biosystems^®^ StepOnePlus^™^ Real‐Time PCR System on 96‐well plates with SensiMix^™^ SYBR^®^ from the No‐ROX Kit as the reporter mix. Fold changes in gene expression were calculated using the 2^−ΔΔCt^ method (Schmittgen & Livak, 2008) modified for amplification efficiencies as per the manufacturer's protocol.

### 2.4 Statistical analysis

Statistical computations were carried out using the data analysis package in SigmaPlot version 12.5, from Systat Software, Inc., San Jose California USA. Survival data were analyzed using the method of Kaplan and Meier (log‐rank test), and differences in mean survival times were analyzed using one‐way analysis of variance (ANOVA). Two‐way ANOVA with interaction effects was used to test for differences in bacterial clearance rates and gene expression levels between photoperiod and treatment groups, and their combinations. Model diagnostics for the ANOVA were also performed and did not reveal any evidence against the model assumptions, such as homoscedasticity and normality of errors.

Holm‐Sidak's method for multiple comparisons was used for post hoc tests of all pairwise differences between combinations of photoperiod and treatment with the control.

## 3 RESULTS

The survival analysis of all four groups of injected larvae demonstrated that control injections had no effect on development or survival given that close to 100% of the control larvae—regardless of the photoperiod regime—reached the pupal stage after 5 days on average. LD control survival was 93.3%, and SD control survival was 100%.

**Figure 1:**
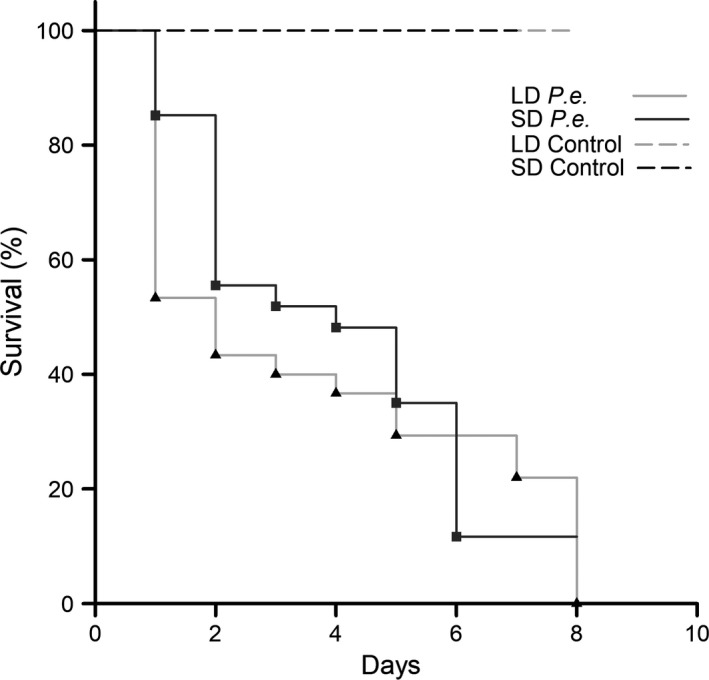
Impact of photoperiod and immune challenge on the mortality of larvae. Dashed lines indicate survivals of long‐day primed (light gray) and short‐day primed (black) controls. Continuous lines indicate survivals of long‐day primed (light gray) and short‐day primed (black) larvae injected with *P. entomophila*. Triangles and squares represent event points in the Kaplan‐Meier diagram

On the other hand, only ~23% of the larvae injected with *P. entomophila* (both the long‐day and short‐day generations) survived until pupation, although these also reached the pupal stage 5–6 days postinjection. Notably, larvae representing the long‐day generation died significantly faster (*p* = .008, F = 4.03, *df* = 50) than those representing the short‐day generation, that is, after only 2.5 days compared to 5 days, a trend which also reflected in the corresponding survival curves (Figure 1). Notwithstanding the treatments, if larvae had not pupated by 9 days postinjection, they invariably died (data not shown).

**Figure 2:**
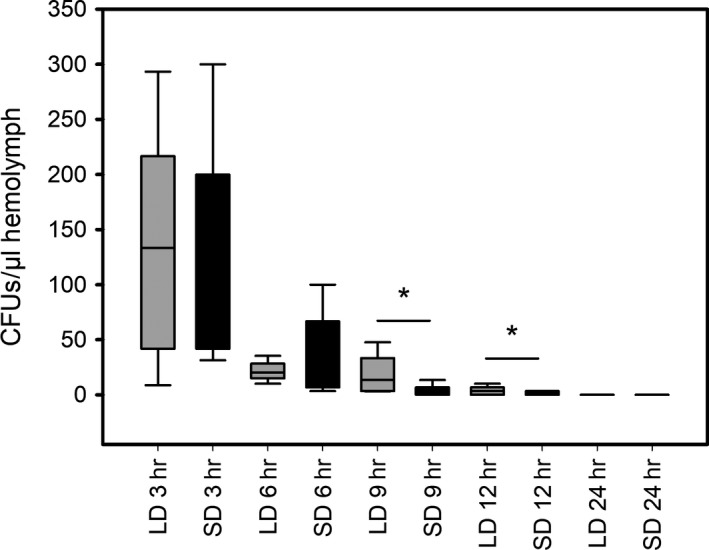
Impact of photoperiod on the bacterial loads of *P. entomophila* in the hemolymph over a one‐day period. Long‐day primed (LD) and short‐day primed (SD) samples are displayed in juxtaposition for individual time points. Black cross lines in bars represent the median values. Error bars represent standard deviations. Asterisks above bars indicate significant differences according to pairwise multiple comparison procedures (Holm‐Sidak method)

The bacterial clearance rates in the larval hemolymph (Figure 2) also differed between the long‐day and short‐day generations at the sampling points 9 and 12 hr postinjection. However, after 1 day, GFP‐marked bacterial pathogens were undetectable in either of the groups. Specifically, by 3 and 6 hr postinjection, both groups had comparably high levels of freely circulating bacteria in the hemolymph which then declined continually over the following sampling points, albeit at different rates. Nine hours after the bacterial challenge, long‐day larvae still had significantly more (*p* < .005, F = 9.4, *df* = 63) circulating bacteria than short‐day larvae (5:1 ratio), and after 12 hr, the difference was still significant (*p* = .053, *F* = 3.9, *df* = 72) with over three times as many bacteria per microliter hemolymph in the long‐day larvae.

**Figure 3:**
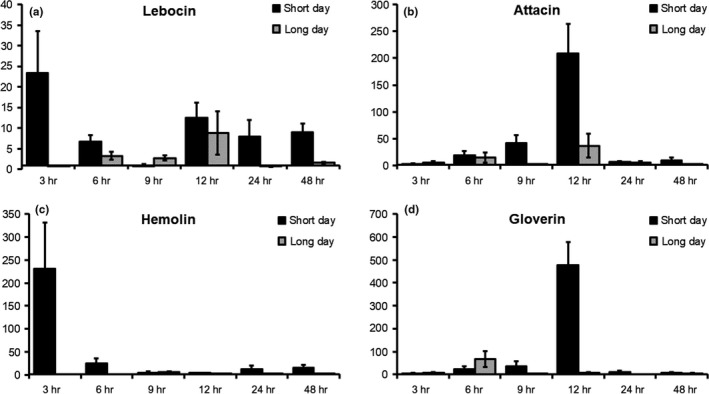
Impact of photoperiod on the expression of immunity‐related genes after an immune challenge over a two‐day period. Long‐day primed (light gray) and short‐day primed (black) treatments are displayed in juxtaposition for individual timepoints. Relative fold changes for each gene were set to 1 for the control treatment and normalized against the Ribosomal protein L10 housekeeping gene. Error bars represent standard deviations. (a) Lebocin, (b) Attacin, (c) Hemolin, (d) Gloverin

The expression levels of examined AMP genes were higher in the short‐day larvae than the long‐day larvae across all sampling points (Figure 3). AMP genes were selected as markers based on their relative expression levels in a recent transcriptome analysis (Vilcinskas & Vogel, 2016) but also the ability of the corresponding AMPs to kill Gram‐negative bacteria such as *P. entomophila*. Interestingly, in short‐day larvae, the four tested AMP genes clustered into two distinct response phases—namely early and delayed expression (Figure 3a,c vs b,d). Specifically, the expression of the lebocin and hemolin genes peaked only 3 hr after the challenge and then returned to medium (lebocin) or very low levels (hemolin) for the remaining sampling points (Figure 3a,c). In contrast, attacin and gloverin gene expression continuously increased from very low levels 3, 6, and 9 hr postinjection to peak strongly after 12 hr and then drop back to even lower levels over the remaining sampling points (Figure 3b,c). In long‐day larvae, only the lebocin and attacin genes showed a comparable—but markedly less pronounced—expression trajectory (Figure 3a,b), whereas hemolin and gloverin were expressed at negligible levels (Figure 3c,d).

## 4 DISCUSSION

The map butterfly *A. levana* is a textbook example of morphological polyphenism due to the strikingly different dorsal wing patterns and more subtle differences in wing size and shape between the spring and summer morphs. Accordingly, most previous studies have focused on morphological polyphenism in the adult, with little attention paid to nonmorphological traits and no reports looking at the larval and pupal stages of development. Here we report for the first time a nonmorphological polyphenism manifesting at the larval stage, reflecting a difference in immunocompetence between larvae photoperiodically primed for subitaneous development (summer generation) and diapause development (spring generation). Specifically, following infection with the entomopathogenic bacterium *P. entomophila*, long‐day larvae died much earlier than their short‐day counterparts (Figure 1), were less efficient at reducing the pathogenic load in the hemolymph (Figure 2), and struggled to mount an efficient AMP‐mediated immune response (Figure 3). In Lepidopterans, all four tested AMPs are known to be active against (at least) Gram‐negative bacteria like *P. entomophila*. An exact mode of action is often unknown but it has been suggested that many AMPs promote the permeability of the bacterial plasma membrane or inhibition of outer‐membrane protein synthesis (Jiang, Vilcinskas, & Kanost, 2010).

These findings agree with studies reporting photoperiodic immunological changes in small mammals, which generally indicated that shorter day lengths are associated with more potent immune responses (Nelson & Demas, 1996). Similarly, Fedorka, Copeland, and Winterhalter (2013) demonstrated that rearing the cricket *Allonemobius socius* under fall‐like conditions resulted in greater resistance to the entomopathogenic bacterium *Serratia marcescens* than summer‐like conditions (Fedorka et al., 2013). However, the authors of the latter study co‐varied the temperature and photoperiod, making it more difficult to determine which of the two cues was the main variable responsible for the observed results.

The explanation for such marked differences in immunocompetence is unclear. Many insects are relatively short lived, so it is advantageous for temporally isolated cohorts to adapt to seasonal changes in the climate and in the available resources. Seasonal polyphenism can therefore be considered an adaptation to consistent and predictable variations in the environment such that each seasonal phenotype achieves the greatest fitness in the environmental condition under which the phenotype evolved (Brakefield, 1996; Nijhout, 2003). The wing polyphenism observed in many lepidopteran species may reflect two major ultimate causes: first, thermoregulation and climate adaptations influence seasonally varying color patterns (Hazel, 2002; Kemp & Jones, 2001); and second, predator avoidance as has been shown in many other lepidopteran species (Marsh & Rothschild, 1974, Wiklund & Tullberg 2004). In *A. levana*, the wing polyphenism probably did not evolve as a thermoregulatory response because the origin of the polyphenism predates the dispersal of the species toward Palearctic temperate regions (Fric et al., 2004). Neither is the predation‐avoidance hypothesis truly compelling (Ihalainen & Lindstedt, 2012). An alternative explanation would be an induction of higher metabolism due to a longer photoperiod, distributing costs to a more elaborate wing color and morphology, thus limiting resources for immunity. On the other hand, a more effective immune system in short‐day morph pupae (cold period) would be beneficial to survive exposition to litter with a high microbial titer. However, currently there is no indication of a functional link between the larval immunity‐related polyphenism reported in this study and the diphenistic wing coloration encountered in adult butterflies. A possible explanation for the difference in juvenile immunocompetence is that immunocompetence is traded off against dispersal and fecundity in the summer generation. Adult members of the summer generation seem to be better adapted to long‐distance flights (through larger thoracic flight musculature), are more active than the spring generation, and invest more resources into reproduction (Friberg & Karlsson, 2010; Fric & Konvička, 2002; Morehouse et al., 2013). All these traits (especially reproduction) are energetically costly, but so is a powerful and active immune system. Life‐history theory therefore predicts that the fitness value of one trait may lower the fitness value of another and a trade‐off between reproduction and immunity has been demonstrated in various insect taxa under many different experimental settings (Schwenke et al., 2016; Siva‐Jothy, Moret, & Rolff, 2005; Vilcinskas, 2013). In line with these findings, in the hemipteran *Acyrthosiphon pisum*, a positive relationship between fecundity and susceptibility to parasitoid attack was found (Gwynn, Callaghan, Gorham, Walters, & Fellowes, 2005). In the same aphid species, it was shown that melanization and fecundity are under control of molecules displaying pleiotropic roles in embryogenesis, longevity, fecundity, and melanization (Will, Schmidtberg, Skaljac, & Vilcinskas, 2017).

The observed Immunological larval polyphenism may result from the crosstalk between pathways regulating transcriptional reprogramming. Particularly epigenetic mechanisms such as histone acetylation and microRNAs link complex traits such as immunity and development in insects (Freitak, Knorr, Vogel, & Vilcinskas, 2012; Mukherjee, Fischer, & Vilcinskas, 2012). It is worth mentioning here, that if we assume an antagonistic pleiotropy for the life‐history traits immunity and melanization, the reduced immunity may be a necessary consequence of the darker summer morph (and vice versa). In fact, both melanin‐based pigmentation and the component of the insects’ immune system known as the melanization cascade rely on, and thus compete for the same precursor resource, namely the amino acid tyrosine. Melanization is known to be costly in insects and for example negatively impacts reproduction investment in the sand cricket, *Gryllus firmus* (Roff & Fairbairn, 2013). Arguably, the immunity versus melanization case represents a classical physiological trade‐off (Dubovskiy et al., 2013). An additional explanation for it may be that the increased pigmentation of the derived summer morph evolved due to increased day lengths in higher latitudes which correspond to an increase in UV light exposure. In that case, the darker summer morph may be a genoprotective response to this new hazard.

Although firmly grounded in the literature, here we admittedly rely on speculation and thus further research is needed to determine whether the immunity‐related polyphenism is maintained in adult *A*. *levana*, if the species indeed displays behavioral thermoregulatory responses to an array of natural pathogens/parasites, and if there is a difference in warm‐up speeds and absolute body temperature through solar radiation absorbance guided by the phenotype. Moreover, it would be interesting to test the nature and relative extent of pathogen/parasite loads in wild spring and summer generations to see if the immunity‐related polyphenism we have discovered is manifested under natural conditions. Future studies should be complemented by an infection assay where both phenotypes are subsequently assessed in parallel for their reproductive outputs.

Our study provides the first indication that wing color morphology in adult *A. levana* is not the only phenotype with a plastic response to day length. We show that the juvenile stages display an immunity‐related polyphenism that may at least partially explain the derived darker‐wing phenotype of the summer generation: Long‐day larvae have a weaker immune response than short‐day larvae, which they potentially compensate through behavioral fever. We acknowledge that immunity is an aspect that is clearly underrepresented in studies concerned with lepidopteran polyphenisms and their ultimate causes, which we have begun to address in this study.

## ACKNOWLEDGMENTS

This work was funded by the excellence initiative of the Hessian Ministry of Science, Higher Education and Art (HMWK) supporting the LOEWE Centre for Insect Biotechnology and Bioresources. The authors thank Dr Richard M. Twyman for professional editing of the manuscript and Prof. Bruno Lemaitre for providing us with the entomopathogen *Pseudomonas entomophila*.

## CONFLICTS OF INTEREST

The authors have no conflicts of interest to declare.

## AUTHOR CONTRIBUTIONS

AB performed the experiments and reared *A. levana*. HV extracted the AMP‐encoding sequences from the transcriptomic data base. KL and AV designed the study. AB, KL, and AV drafted the manuscript. AV provided funding.
