# Supplementary material for: Immunological larval polyphenism in the map butterfly Araschnia levana reveals the photoperiodic modulation of immunity
Source: Ecol Evol. 2018 Apr 19;8(10):4891–8. doi: 10.1002/ece3.4047 (PMC5980286; doi:10.1002/ece3.4047)
Supplement: Supplementary file 1 [file ECE3-8-4891-s001.docx]

**Immunological larval polyphenism in the map butterfly *Araschnia levana* reveals the photoperiodic modulation of immunity**

**Arne Baudach^1^, Kwang-Zin Lee^1^, Heiko Vogel^2^ & Andreas Vilcinskas^1,3^**

**^1^**Institute for Insect Biotechnology, Justus Liebig University, Heinrich Buff Ring 26-32, 35392 Giessen, Germany

^2^Max-Planck Institute for Chemical Ecology, Hans Knoell Strasse 8, 07749 Jena, Germany

^3^Fraunhofer Institute for Molecular Biology and Applied Ecology, Department Bioresources, Winchesterstrasse 2, 35394 Giessen, Germany

**Supplement**

**Table 1 – Primer sequences**

| **Supplemental Table 1.** *A. levana* primer sequences for the AMP genes and the housekeeping gene used for normalization. | | |  |  |
| --- | --- | --- | --- | --- |
|  |  |  |  |  |
|  | **qRT-PCR Primer Sequences** | |  |  |
|  |  |  |  |  |
| **Gene** | **Forward Primer** | **Reverse Primer** |  |  |
| Attacin | ATGGAGGAGGTGTTGATTACAGC | TTAGTAGCACCAGCGTTGAAGTC |  |  |
| Gloverin | GCCTCGGTAACACGGAAGATG | TCGCCACTTGGACTGAGAACTC |  |  |
| Hemolin | CAATTCGTGAGGGACCATCTAC | TTATTCTATCGCTGTGAGGCAAG |  |  |
| Lebocin | CAGCCAGAATATTGTTTGAAAGTC | AGCGAAGGAAATTATGCTCTTTG |  |  |
| Ribosomal protein L10 | GGCAAGGACCAGTTTCACAT | CCAGTCTGGAGCCTATCAGC |  |  |
